# Physapruin A Exerts Endoplasmic Reticulum Stress to Trigger Breast Cancer Cell Apoptosis via Oxidative Stress

**DOI:** 10.3390/ijms24108853

**Published:** 2023-05-16

**Authors:** Tzu-Jung Yu, Jun-Ping Shiau, Jen-Yang Tang, Ammad Ahmad Farooqi, Yuan-Bin Cheng, Ming-Feng Hou, Chia-Hung Yen, Hsueh-Wei Chang

**Affiliations:** 1 Graduate Institute of Natural Products, Kaohsiung Medical University, Kaohsiung 80708, Taiwan; u109831002@kmu.edu.tw; 2 Division of Breast Oncology and Surgery, Department of Surgery, Kaohsiung Medical University Hospital, Kaohsiung Medical University, Kaohsiung 80708, Taiwan; 1060526@kmuh.org.tw (J.-P.S.); mifeho@kmu.edu.tw (M.-F.H.); 3 School of Post-Baccalaureate Medicine, Kaohsiung Medical University, Kaohsiung 80708, Taiwan; reyata@kmu.edu.tw; 4 Department of Radiation Oncology, Kaohsiung Medical University Hospital, Kaohsiung Medical University, Kaohsiung 80708, Taiwan; 5 Institute of Biomedical and Genetic Engineering (IBGE), Islamabad 54000, Pakistan; farooqiammadahmad@gmail.com; 6 Department of Marine Biotechnology and Resources, National Sun Yat-sen University, Kaohsiung 80424, Taiwan; jmb@mail.nsysu.edu.tw; 7 Department of Biomedical Science and Environmental Biology, College of Life Science, Kaohsiung Medical University, Kaohsiung 80708, Taiwan; 8 National Natural Product Libraries and High-Throughput Screening Core Facility, Kaohsiung Medical University, Kaohsiung 80708, Taiwan; 9 Center for Cancer Research, Kaohsiung Medical University, Kaohsiung 80708, Taiwan

**Keywords:** withanolide, ER expansion, aggresome, caspase activation, *N*-acetylcysteine, ROS

## Abstract

*Physalis* plants are commonly used traditional medicinal herbs, and most of their extracts containing withanolides show anticancer effects. Physapruin A (PHA), a withanolide isolated from *P. peruviana*, shows antiproliferative effects on breast cancer cells involving oxidative stress, apoptosis, and autophagy. However, the other oxidative stress-associated response, such as endoplasmic reticulum (ER) stress, and its participation in regulating apoptosis in PHA-treated breast cancer cells remain unclear. This study aims to explore the function of oxidative stress and ER stress in modulating the proliferation and apoptosis of breast cancer cells treated with PHA. PHA induced a more significant ER expansion and aggresome formation of breast cancer cells (MCF7 and MDA-MB-231). The mRNA and protein levels of ER stress-responsive genes (*IRE1α* and *BIP*) were upregulated by PHA in breast cancer cells. The co-treatment of PHA with the ER stress-inducer (thapsigargin, TG), i.e., TG/PHA, demonstrated synergistic antiproliferation, reactive oxygen species generation, subG1 accumulation, and apoptosis (annexin V and caspases 3/8 activation) as examined by ATP assay, flow cytometry, and western blotting. These ER stress responses, their associated antiproliferation, and apoptosis changes were partly alleviated by the *N*-acetylcysteine, an oxidative stress inhibitor. Taken together, PHA exhibits ER stress-inducing function to promote antiproliferation and apoptosis of breast cancer cells involving oxidative stress.

## 1. Introduction

Breast cancer was one of the most prevalent female cancer types for new cases and deaths worldwide, as reported by Cancer Statistics in 2023 [1]. Most breast cancer patients differentially expressed three common biomarkers, i.e., estrogen receptor (ER), human epidermal growth factor receptor 2 (HER2), and progesterone receptor (PR) [2]. The remaining cancer patients (10–20%) show a negative expression for these three biomarkers, namely triple-negative breast cancer (TNBC) [2,3]. Target therapy for non-TNBC is mature, such as ER [4], HER2 [5], and PR [6] specific treatments. Notably, these target therapies for non-TNBC are ineffective for TNBC due to the negative expressions of these biomarkers, leading to a higher cell death rate in TNBC than in non-TNBC [7,8]. Moreover, the side effects [9] and drug resistance [10] of chemotherapy for breast cancer are frequently unavoidable. Accordingly, the anticancer drug discovery for TNBC and non-TNBC is still urgently needed.

*Physalis* plants, traditional medicinal herbs used in Asia and South America [11], contain withanolides, the C-28 steroidal lactones [11], and show anticancer effects [12,13,14,15,16,17]. Physapruin A (PHA), a withanolide isolated from *P. peruviana*, was first reported in 1933 [18] and showed antiproliferation effects for human prostate, renal [19], oral [20], and breast [21] cancer cells recently. The function of oxidative stress and apoptosis has been validated in these oral and breast cancer studies [20,21]. However, PHA’s anticancer effects and mechanisms still warrant a detailed investigation, particularly for other oxidative stress-associated functions.

Endoplasmic reticulum (ER) functions include protein synthesis, folding, lipid metabolism, calcium homeostasis, and cell stress responses to drugs [22,23]. When cells suffer from vast amounts of oxidative stress, the protein misfolding and aggregating become severe and expand the ER [24], namely ER stress [25]. Several ER stress-responsive signaling has been reported. For example, heat-shock protein family A member 5 (BIP) is a central ER stress modulator connecting to three kinds of unfolded-protein-response proteins, such as protein kinase RNA-like ER kinase (PERK), inositol-requiring enzyme 1 alpha (IRE1α), and activating transcription factor 6 (ATF6) [26].

Although PHA can promote oxidative stress in breast cancer cells [21], the potential of ER stress on breast cancer cells remains unclear. This study aims to evaluate the ER stress response and mechanism acting on the antiproliferative effects of breast cancer cells. Moreover, the function of oxidative stress on the PHA-modulated ER stress is also investigated.

## 2. Results

### 2.1. PHA Promotes ER Expansion

**Figure 1:**
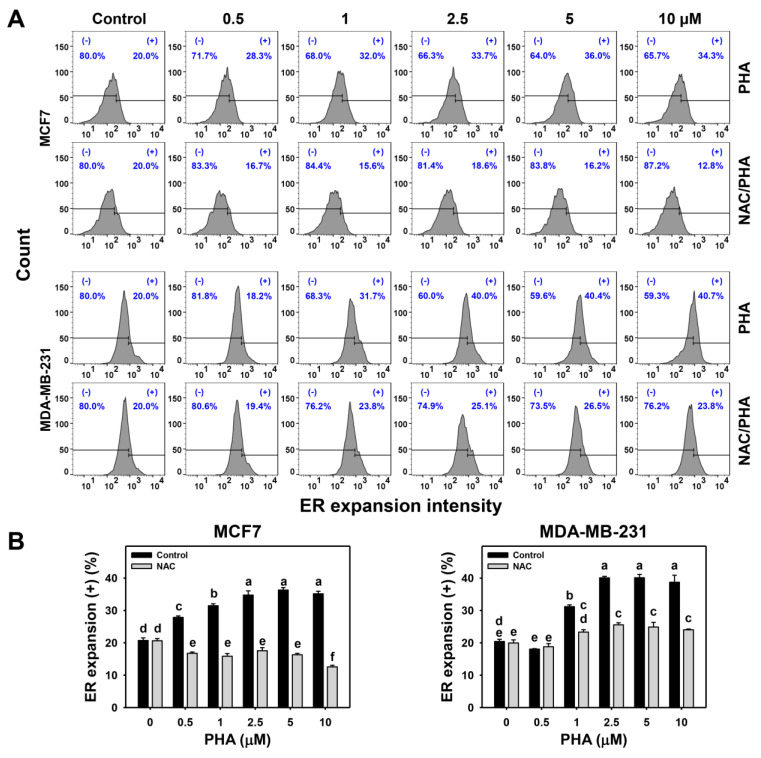
PHA promotes ER expansion in breast cancer cells. (**A**,**B**) Pattern and quantification of ER expansion analysis. After NAC pretreatment, cells were treated with 0 (0.1% DMSO), 0.5, 1, 2.5, 5, and 10 μM PHA for 24 h. (+) proportion was marked for ER expansion (+) (%). For multiple comparisons, treatments displaying nonoverlapped letters have significant results of the same cell lines (*p* < 0.05) based on One-way ANOVA and Turkey HSD Post-Hoc Tests.

After treating with PHA (control, 0.5, 1, 2.5, 5, and 10 μM), the ER expansion intensity was determined by flow cytometry (Figure 1A). PHA upregulated ER expansion (+) (%) of breast cancer cells (MCF7 and MDA-MB-231) in a dose-response manner (Figure 1B), alleviated by *N*-acetylcysteine (NAC) pretreatment (ROS inhibitor). This result suggests PHA promotes ER expansion of breast cancer cells mediated by ROS.

### 2.2. PHA Promotes Aggresome Formation

**Figure 2:**
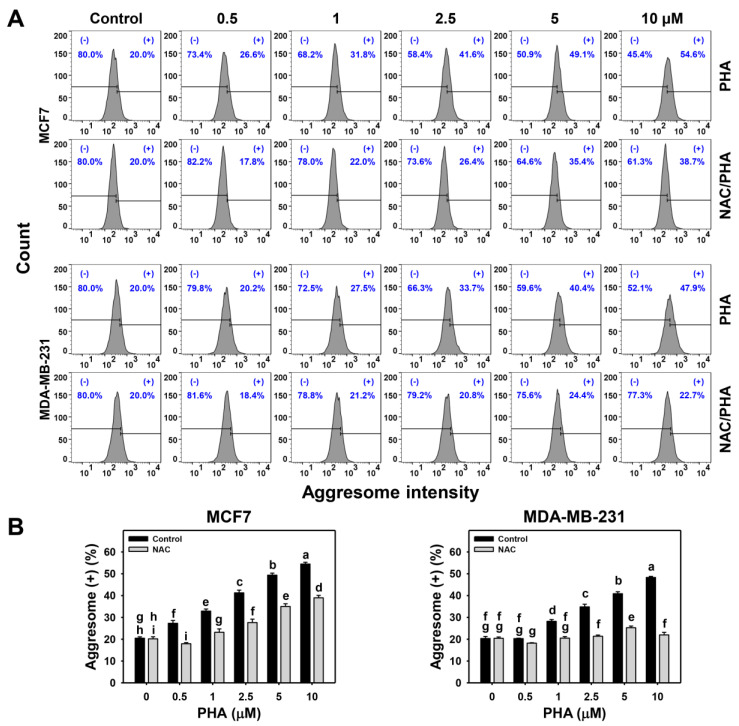
PHA promotes aggresome formation in breast cancer cells. (**A**,**B**) Pattern and quantification of aggresome formation analysis. After NAC pretreatment, cells were treated with 0 (0.1% DMSO), 0.5, 1, 2.5, 5, and 10 μM PHA for 24 h. (+) proportion was marked for aggresome aggregation (+) (%). For multiple comparisons, treatments displaying nonoverlapped letters have significant results of the same cell lines (*p* < 0.05) based on One-way ANOVA and Turkey HSD Post-Hoc Tests.

ER exhibits protein folding and delivery functions. The accumulation of misfolded or denatured proteins causes aggresome formation, contributing to ER stress [27]. After treating with PHA (control, 0.5, 1, 2.5, 5, and 10 μM), the aggresome intensity was determined by flow cytometry (Figure 2A). PHA upregulated aggresome (+) (%) of breast cancer cells (MCF7 and MDA-MB-231) in a dose-response manner (Figure 2B). This PHA-induced aggresome formation was alleviated by NAC pretreatment in MDA-MB-231 cells but was partly decreased by NAC in MCF7 cells. This result suggests PHA promotes the aggresome formation of breast cancer cells partly mediated by ROS.

### 2.3. PHA Upregulates ER Stress-Responsive Gene Expressions

**Figure 3:**
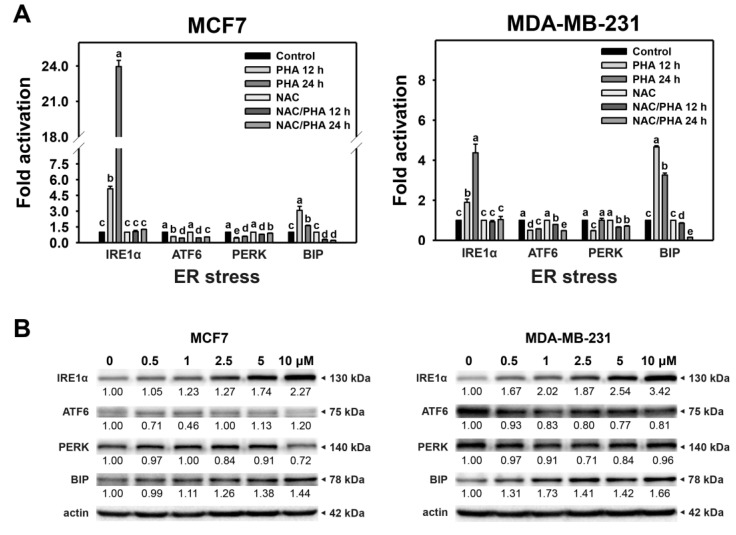
PHA promotes mRNA and protein expressions of ER stress-responsive genes in breast cancer cells. (**A**) Fold activation of mRNA expression for *IRE1α*, *ATF6*, *PERK*, and *BIP* genes. After NAC pretreatment, cells were treated with 0 (0.1% DMSO) and 10 μM PHA for 12 and 24 h, namely NAC, NAC/PHA 12 h, and NAC/PHA 24 h. Except for NAC, the treatments for PHA only were the same as NAC/PHA treatment, i.e., control, PHA 12 h, and PHA 24 h. For multiple comparisons of the same gene, treatments displaying nonoverlapped letters have significant results of the same cell lines (*p* < 0.05) based on One-way ANOVA and Turkey HSD Post-Hoc Tests. (**B**) Western blotting for ER stress-responsive genes (IRE1α, ATF6, PERK, and BIP). Cells were treated with 0 (0.1% DMSO), 0.5, 1, 2.5, 5, and 10 μM PHA for 24 h. The values under bands indicate the relative protein expression compared with the control β-actin.

The mRNA and protein levels of ER stress-responsive genes, such as *IRE1α*, *ATF6*, *PERK*, and *BIP*, were monitored by qRT-PCR (Figure 3A) and western blotting (Figure 3B). The relative mRNA expressions of *IRE1α* and *BIP* genes were higher in PHA-treated breast cancer (MCF7 and MDA-MB-231) cells than in control (Figure 3A), alleviated by NAC pretreatment. In comparison, the mRNA levels of ATF6 and PERK genes were weakly changed.

Similarly, ER stress-responsive proteins of PHA-treated breast cancer cells were assessed (Figure 3B). In MCF7 and MDA-MB-231 cells, PHA moderately upregulated IRE1α and BIP protein expressions compared to the control. In comparison, ATF6 was slightly regulated by PHA in MCF7 but not MDA-MB-231 cells. While PERK gene protein levels were weakly changed in both cell lines.

### 2.4. TG Promotes PHA-Induced ROS Generation

**Figure 4:**
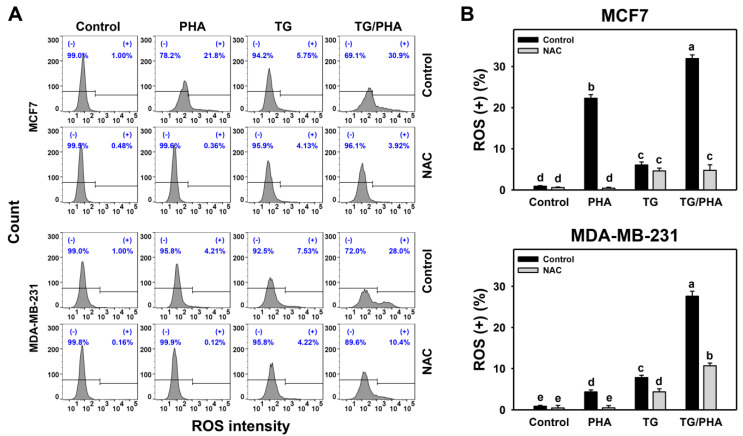
Thapsigargin (TG) promoted PHA-induced ROS generation in breast cancer cells. (**A**,**B**) Pattern and quantification of ROS analysis. After NAC pretreatment, cells were treated with 0 (0.1% DMSO), PHA (2.5 μM), TG (0.1 μM), or TG/PHA for 24 h. TG/PHA is the co-treatment of TG (0.1 μM)/PHA (2.5 μM). (+) proportion was marked for high ROS (+) (%). For multiple comparisons, treatments displaying nonoverlapped letters have significant results of the same cell lines (*p* < 0.05) based on One-way ANOVA and Turkey HSD Post-Hoc Tests.

ROS effects on ER stress response and signaling (Figure 1, Figure 2 and Figure 3) were validated by NAC. It warrants an assessment of the ROS response of PHA and the impact of ER stress on ROS changes in breast cancer cells (Figure 4A). Individual PHA and thapsigargin (TG) treatments upregulated the ROS generation in breast cancer cells (Figure 4B). Moreover, TG/PHA co-treatment showed more significant ROS generation than PHA or TG, suggesting TG/PHA causes synergistic ROS generation. Furthermore, ROS induction exerted by PHA and/or TG was partly alleviated by NAC pretreatment except for TG treatment in MCF7 cells. This further validated the ROS-inducing ability of PHA and/or TG in breast cancer cells.

### 2.5. TG/PHA Co-Treatment Caused Synergistic Antiproliferation

**Figure 5:**
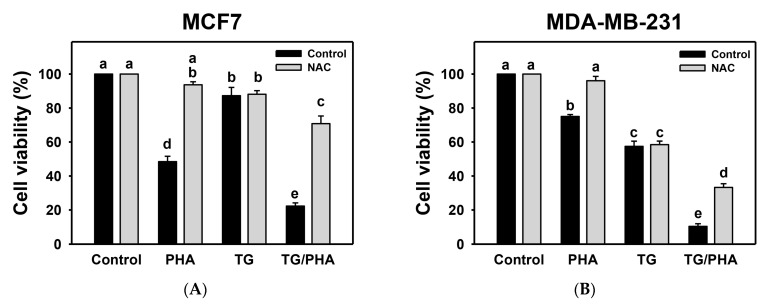
TG promoted PHA-induced antiproliferation in breast cancer cells. (**A**,**B**) ATP-based cell viability. After NAC pretreatment, cells were treated with 0 (0.1% DMSO), PHA (2.5 μM), TG (0.1 μM), or TG/PHA for 24 h. TG/PHA is the co-treatment of TG (0.1 μM)/PHA (2.5 μM). For multiple comparisons, treatments displaying nonoverlapped letters have significant results of the same cell lines (*p* < 0.05) based on One-way ANOVA and Turkey HSD Post-Hoc Tests.

Since ROS is a proliferation-modulator [28], the impact of proliferation-modulating effects of PHA and/or TG warrants a detailed investigation. TG/PHA co-treatment of breast cancer cells decreased cell viability by 22.27% and 10.41% compared to separate treatments (PHA or TG) in MCF7 (48.46% or 87.27%) and MDA-MB-231 (75.06% or 57.46%) cells, respectively (Figure 5).

Moreover, the potential role of ROS in the proliferation-modulating effects of TG/PHA co-treatment was validated by NAC pretreatment. NAC/TG/PHA treatment showed higher cell viability than TG/PHA co-treatment in breast cancer cells. The α values of TG/PHA in breast cancer cells for the ATP assay (MCF7 vs. MDA-MB-231) were 1.90 ± 0.09 and 4.20 ± 0.66, respectively. This suggests that TG/PHA exerts synergistic antiproliferation against breast cancer cells involving ROS regulation.

### 2.6. TG/PHA Co-Treatment Caused Synergistic SubG1 Accumulation

**Figure 6:**
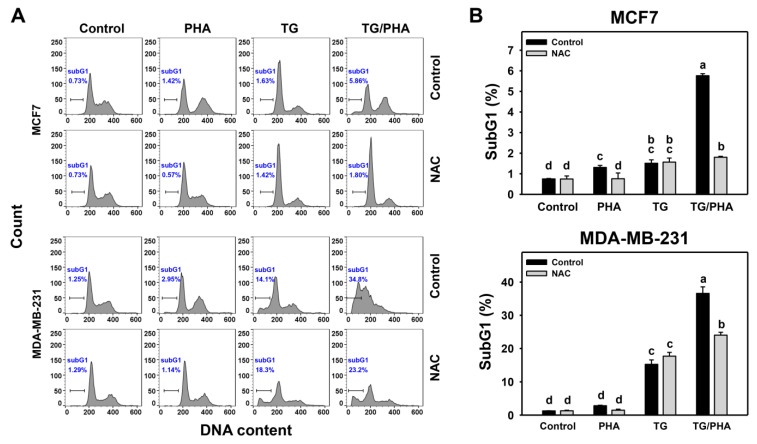
TG promoted PHA-induced subG1 accumulation in breast cancer cells. (**A**,**B**) Pattern and quantification of cell cycle analysis. After NAC pretreatment, cells were treated with 0 (0.1% DMSO), PHA (2.5 μM), TG (0.1 μM), or TG/PHA for 24 h. TG/PHA is the co-treatment of TG (0.1 μM)/PHA (2.5 μM). The subG1 proportion was marked for subG1 (%). For multiple comparisons, treatments displaying nonoverlapped letters have significant results of the same cell lines (*p* < 0.05) based on One-way ANOVA and Turkey HSD Post-Hoc Tests.

Since ROS is a cell cycling modulator [29], the impact of cell cycle progression-modulating effects of PHA and/or TG warrants a detailed investigation (Figure 6A). TG/PHA co-treatment of breast cancer cells showed a more significant proportion of subG1% compared to separate treatments (PHA or TG) in breast cancer cells (Figure 6B).

Moreover, the potential function of ROS in subG1 change of TG/PHA co-treatment was validated by NAC pretreatment. NAC/TG/PHA treatment showed lower subG1% than TG/PHA co-treatment in breast cancer cells. This suggests that TG/PHA exerts synergistic subG1 accumulation against breast cancer cells involving ROS regulation.

### 2.7. TG/PHA Co-Treatment Caused Synergistic Apoptosis

**Figure 7:**
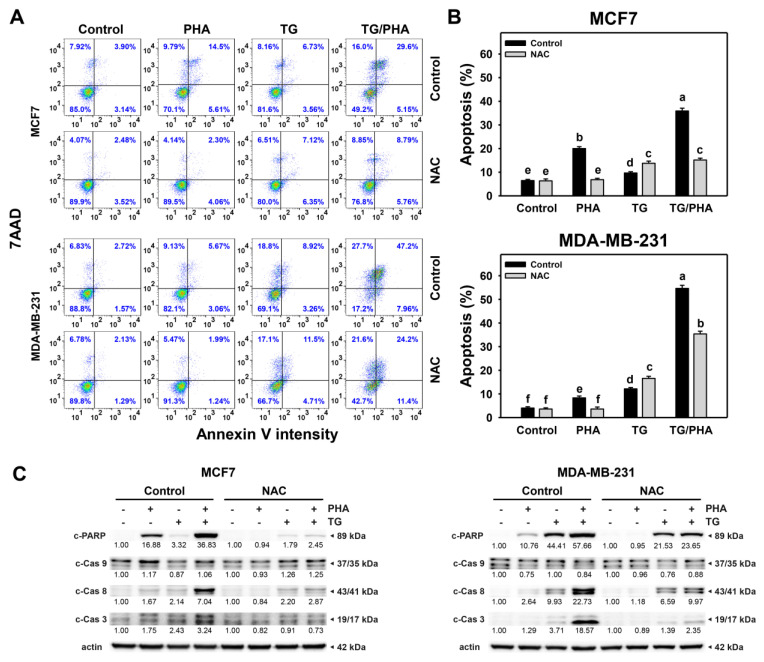
TG promoted PHA-induced apoptosis in breast cancer cells. (**A**,**B**) Pattern and quantification of annexin V/7AAD analysis. After NAC pretreatment, cells were treated with 0 (0.1% DMSO), PHA (2.5 μM), TG (0.1 μM), or TG/PHA for 24 h. TG/PHA is the co-treatment of TG (0.1 μM)/PHA (2.5 μM). Annexin V (+)/7AAD (+)/(−) proportions were counted for apoptosis (+) (%). For multiple comparisons, treatments displaying nonoverlapped letters have significant results of the same cell lines (*p* < 0.05) based on One-way ANOVA and Turkey HSD Post-Hoc Tests. (**C**) Western blotting for apoptosis genes (cleaved PARP (c-PARP), c-Cas 3, c-Cas 8, and c-Cas 9). The values under bands indicate the relative protein expression compared with the control β-actin.

Since ROS is an apoptosis-modulator [30], the impact of apoptosis-modulating effects of PHA and/or TG warrants a detailed investigation (Figure 7A). TG/PHA co-treatment of breast cancer cells showed a more significant proportion of annexin V (+)% compared to separate treatments (PHA or TG) in breast cancer cells (Figure 7B).

Moreover, the potential function of ROS in annexin V change of TG/PHA co-treatment was validated by NAC pretreatment that NAC/TG/PHA treatment showed lower annexin V (+)% than TG/PHA co-treatment in breast cancer cells. For the western blotting (Figure 7C), TG/PHA co-treatment showed a higher expression of apoptotic proteins such as c-PARP, c-Cas 3, and c-Cas 8 compared to separate treatments (TG or PHA) in breast cancer cells. In contrast, c-Cas 9 showed little changed in TG and/or PHA. Moreover, these TG/PHA-triggered apoptotic protein expressions were suppressed by NAC pretreatment. This suggests that TG/PHA exerts synergistic apoptosis (annexin V) and apoptotic signaling against breast cancer cells involving ROS regulation.

## 3. Discussion

The anticancer effects and mechanism of PHA were sporadically investigated. Literature report [19] focused on the isolation of bioactive compounds from *P. peruviana* and provided only the cytotoxicity of prostate and renal cancer cells, lacking detailed mechanisms. Our previous works showed the precise antiproliferation mechanisms of cancer cells, such as the promoting effects of oxidative stress, apoptosis, DNA damage [21], and protective autophagy [31] on breast cancer cells and the DNA repair-suppressing effects on oral cancer cells [20]. These PHA-associated mechanisms share the crucial role of oxidative stress for anticancer effects. Although oxidative stress also triggers ER stress; however, the involvement of ER stress in PHA-induced antiproliferation of breast cancer cells was not reported. Moreover, ER stress also participates in the development of apoptosis [32,33] and autophagy [34,35] in cancer cells. It warrants a thoughtful assessment of ER stress in PHA-treated breast cancer cells.

The ER stress induction was validated by the flow cytometry evidence that ER expansion and aggresome formation were induced in breast cancer cells (Figure 1 and Figure 2). Moreover, this ER stress phenomenon was partly alleviated by NAC. Thus, the role of oxidative stress was confirmed in PHA-triggered ER stress in breast cancer cells. These ER stress responses were further evaluated by examining the ER stress-responsive signaling in mRNA and protein expressions for *IRE1α*, *ATF6*, *PERK*, and *BIP* genes (Figure 3). BIP is a central ER stress regulator that modulates the response of PERK, IRE1α, and ATF6 [26]. Although ER stress is induced, the expressions of *IRE1α*, *ATF6*, *PERK*, and *BIP* genes were not all upregulated. *IRE1α* and *BIP* genes were upregulated in both mRNA and protein levels in breast cancer cells. In contrast, the *PERK* gene was minor changed in both mRNA and protein levels. IRE1α can enhance the mRNA splicing of the X-box binding protein 1 (XBP1) gene [36]. It warrants a detailed assessment of the function of IRE1α in PHA-induced ER stress of breast cancer cells by introducing the IRE1α inhibition strategy in the future. Additionally, the ATF6 mRNA expression is minor in both MCF7 and MDA-MB-231 cells. While ATF6 protein expression is upregulated in MCF7 cells but minor downregulated in MDA-MB-231 cells. These differential expressions between mRNA and protein levels may be attributed to the nature of cell lines, i.e., MCF7 cells are non-TNBC and MDA-MB-23 cells are TNBC.

Oxidative stress can induce ER stress [24,25,37,38]. ROS and ER stress-modulating drugs can improve the anticancer effects [39,40]. The impact of ER stress in PHA-treated breast cancer cells was further assessed by the co-treatment with TG, an ER stress enhancer. ER stress showed antiproliferation in several cancer cells [41,42,43,44]. For example, TG inhibits breast cancer cell proliferation, and the co-treatment of TG with epidermal growth factor-proteolytic A subunit further improves the TG-induced antiproliferative function [41].

Similarly, PHA induced ER stress and decreased cell viability. Moreover, TG/PHA synergistically inhibited the cell viability of breast cancer cells (MCF7 and MDA-MB-231) (Figure 5). These results suggest ER stress is crucial in suppressing breast cancer cell proliferation. This ER stress-mediated antiproliferation of PHA or TG/PHA is partly alleviated by NAC pretreatment. Moreover, the participation of oxidative stress is validated by the ROS upregulation in PHA-treated breast cancer cells (Figure 4), partly alleviated by NAC pretreatment. These findings indicate that oxidative stress is responsible for the ER stress effect of PHA.

Moreover, ER stress and oxidative stress can modulate the apoptosis of cancer cells [45,46]. The apoptosis response in ER stress-mediated antiproliferative warrants a detailed assessment. PHA can induce apoptosis, as evidenced by subG1 accumulation, annexin V increment, PARP cleavage, and caspase 3/8 activation in breast cancer cells (Figure 6 and Figure 7). TG also triggers apoptosis of several cancers, such as adrenocortical carcinoma [42] and prostate [43]. Similarly, TG/PHA triggered more apoptosis than PHA or TG, partly alleviated by NAC. Moreover, extrinsic caspases 8 rather than intrinsic 9 was validated to participate in ER stress-mediated apoptosis of breast cancer cells.

PHA showed different sensibility to the ER stress of the two cell lines (MDA-MB-231 and MCF7). At 0.1 μM treatment, TG inhibited cell viability and induced subG1 accumulation in MDA-MB-231 more than in MCF7 cells (Figure 5 and Figure 6). TG also induced more c-PARP, c-caspase 8, and c-caspase 3 expressions in MDA-MB-231 than in MCF7 cells (Figure 7). Similarly, the co-treatment (TG/PHA) exerted overexpression of c-PARP, c-caspase 8, and c-caspase 3 in MDA-MB-231 more than in MCF7 cells. Although these apoptotic signaling expressions were partly suppressed by NAC, they still kept moderate levels in MDA-MB-231 cells more than in MCF7 cells. Accordingly, the TG/PHA-induced apoptosis is not dependent on ROS increase alone.

As mentioned above, the ER stress induced by PHA is crucial for the observed effect on proliferation and apoptosis. However, no experiments demonstrating the correlation between ER stress induction and cell viability or apoptosis were performed in the present study. Considering the results in Figure 6 and Figure 7, it could be hypothesized that the ER stress induction is an event involved in antiproliferative induction caused by PHA but alone is insufficient to exert the anti-cancer effect and probably is not the only pathway modulated by PHA. Notably, PHA clearly induces cell cycle arrest, an effect that is not evidenced when cells are treated with TG. The possible involvement of other non-ER stress mechanisms of action of PHA also could explain the higher effects obtained with the combined treatment (PHA/TG). Because PHA induced the BIP and IRE1α in breast cancer cells (Figure 3), their inhibitors, such as HA15 [47] and KIRA6 [48], may be applied to understand how much this modulation is important in the PHA effects and support the hypothesis of other non-ER stress pathways involved. Notably, we previously reported that PHA could induce ROS-mediated non-ER stress mechanisms such as DNA damage [21] and autophagy [31] in breast cancer cells and inhibit DNA repair in oral cancer cells [20]. Therefore, it warrants a detailed assessment of the participation of ER stress that interplays with DNA damage, DNA repair, and autophagy using ER stress inhibitors in the future.

## 4. Materials and Methods

### 4.1. Cell Culture

Two breast cancer cell lines (MCF7 and MDA-MB-231) were selected from ATCC (Manassas, VA, USA). They were cultured with a mixed DMEM/F12 medium (3:2) containing 10% bovine serum and P/S antibiotics (Gibco, Grand Island, NY, USA).

### 4.2. Drug Treatments

PHA (BioBioPha Co., Yunnan, China) and ER stress-inducer (thapsigargin, TG) (Sigma-Aldrich, St. Louis, MO, USA) were dissolved in DMSO. *N*-acetylcysteine (NAC) (Sigma-Aldrich) [49,50,51,52,53] was dissolved in PBS. NAC was pretreated with 10 mM for 1 h before drug treatments, such as PHA only (0, 0.5, 1, 2.5, 5, and 10 μM) or PHA (2.5 μM)/TG (0.1 μM) co-treatment. NAC after preincubation was left on the cells. DMSO concentration was adjusted to the same (0.1%) for all treatments.

### 4.3. ER Expansion Detection Assay

The seeding density was 1.5 × 10^5^ (MCF7) or 2 × 10^5^ (MDA-MB-231) cells/well in a 6-well plate. After overnight culture, cells were treated with various concentrations of PHA for 24 h. After harvested by 0.05% Trypsin-EDTA (Gibco, Grand Island, NY, USA), organelle-ID RGB^®^ III dye (Enzo Life Sciences, Farmingdale, NY, USA) [54,55] was used to detect ER contents, which is proportional to the degree of ER stress. When cells are under ER stress, ER contents are increased, called ER expansion. ER detection dye was added to cell suspensions (30 min, 4 °C). After twice washing with culture medium, cells were placed in an incubator for 30 min and then conducted with flow cytometry (Guava easyCyte, Luminex, Austin, TX, USA) using the Red-B channel with the plotter software FlowJo (Becton-Dickinson, Franklin Lakes, NJ, USA).

### 4.4. Aggresome Detection Assay

The seeding density and culture condition are the same as in Section 4.3. Proteostat^®^ Aggresome Detection kit (Enzo Life Sciences) [55,56] was used to detect aggresome levels proportional to the degree of ER stress. Following 4% paraformaldehyde fixation for 30 min, cells were treated with 0.5% Triton X-100 (30 min, 4 °C). Then, an aggresome detection dye (1:10,000) was added to cell suspensions (30 min, RT). After PBS washing, cells were conducted with flow cytometry (Guava easyCyte) using the Red-B channel.

### 4.5. mRNA Expression of ER Stress-Responsive Genes

The seeding density was 4 × 10^5^ (MCF7) or 5 × 10^5^ (MDA-MB-231) cells/60 mm dish. After overnight culture, cells were treated with PHA (10 μM) for 24 h. RNA and cDNA conversion were prepared, and a touch-down program was run for quantitative RT-PCR (qRT-PCR) as described [57]. ER stress-responsive genes such as *IRE1α*, *ATF6*, *PERK*, and *BIP* [58] and internal control *GAPDH* were selected, and their primer information has been reported [55]. The relative mRNA expression was presented as fold activation using the 2^−ΔΔCt^ method [59].

### 4.6. Protein Expression of ER Stress-Responsive Genes

The seeding density was 1 × 10^6^ (MCF7) or 1.5 × 10^6^ (MDA-MB-231) cells/100 mm dish. After overnight culture, cells were treated with various concentrations of PHA for 24 h. ER stress-responsive protein expressions were conducted by western blotting. Antibodies against ATF6 (Abcam; 1:500), IRE1α, PERK, BIP (Cell signaling, 1:1000), and β-actin (Sigma-Aldrich; 1:5000) were applied as described [55].

### 4.7. ROS Assay

The seeding density and culture condition are the same as in Section 4.3. Cells were treated with PHA (2.5 μM) and/or TG (0.1 μM) for 24 h. 10 μM of 2′,7′-dichlorodihydrofluorescein diacetate (H_2_DCF-DA) (Sigma-Aldrich), a ROS-detecting dye, was incubated with cells (30 min, 37 °C), as described [60]. Cells were harvested by 0.05% Trypsin-EDTA. After PBS washing, cells were conducted with flow cytometry (Guava easyCyte) using the Green-B channel.

### 4.8. Cell Viability and Synergy Calculation

The seeding density was 4000 (MCF7) or 6000 (MDA-MB-231) cells/well in a 96-well plate. After overnight culture, cells were treated with PHA (2.5 μM) and/or TG (0.1 μM) for 24 h. Cellular ATP level, a sensitive cell viability assay, was determined by ATPlite Luminescence reagent (PerkinElmer Life Sciences, Boston, MA, USA) [55]. The antiproliferation synergy (α) of the co-treatment (TG/PHA) was assessed as follows [61,62]: α = survival fraction (TG) × survival fraction (PHA)/survival fraction (TG/PHA). α > 1, =1, and <1 represent synergistic, additive, or antagonistic effects, respectively.

### 4.9. Cell Cycle Assays

The seeding density and culture condition are the same as in Section 4.3. Cells were harvested by 0.05% Trypsin-EDTA. 5 μg/mL of 7-aminoactinmycin D (7AAD) (Biotium, Hayward, CA, USA), a DNA dye, was used to stain the DNA content of 70% ethanol-fixed cells for 30 min [63]. After PBS washing, cells were conducted with flow cytometry (Guava easyCyte) using the Red-B channel.

### 4.10. Apoptosis Analysis (Annexin V Flow Cytometry and Western Blotting)

The seeding density and culture condition are the same as in Section 4.3. Cells were treated with PHA (2.5 μM) and/or TG (0.1 μM) for 24 h. Annexin V-FITC (1:1000 dilution) (Strong Biotech Corporation, Taipei, Taiwan)/7AAD (1 μg/mL), the double staining reagents, were incubated with cells (30 min, 37 °C) [64]. After PBS washing, cells were conducted with flow cytometry (Guava easyCyte) using the Green-B/Red-B channels. Moreover, the apoptotic protein expressions after drug treatment were assessed by western blottings, such as cleaved types of poly (ADP-ribose), polymerase (c-PARP) and caspases 3, 8, and 9 (c-Cas 3, 8, and 9) (Cell signaling), accompanied by β-actin control (Sigma-Aldrich).

### 4.11. Statistical Analysis

One-way ANOVA and Turkey HSD Post-Hoc Tests was applied to determine significance using JMP^®^12 software (JMP 12 software, SAS Institute Inc., Cary, NC, USA). In multiple comparisons, treatments showing non-overlapping low cases have a significant outcome. Data were shown as means ± SDs (*n* = 3).

## 5. Conclusions

Previously, we have demonstrated that PHA exhibits oxidative stress-inducing functions for apoptosis [21] and cytoprotective autophagy [31] in breast cancer cells. Moreover, oxidative stress can trigger other functions, such as ER stress, but the role of ER stress in PHA-treated breast cancer cells remains unexplored. The present study validated the ER stress responses of PHA by finding ER stress expansion and aggresome formation using flow cytometry. Moreover, this ER stress induction was further confirmed by upregulating ER stress-responsive *IRE1α* and *BIP* genes in both mRNA and protein levels in breast cancer cells.

The role of PHA-promoted ER stress was confirmed to participate in antiproliferation, apoptosis, and extrinsic caspase signaling in breast cancer cells by the TG (ER stress enhancer)/PHA co-treatment experiments in terms of cell viability, subG1 accumulation, annexin V-detected apoptosis, and caspase 3/8 activation. All these ER stress responses and proliferation/apoptosis-modulating effects on breast cancer cells were validated to depend on oxidative stress by NAC pretreatment. Therefore, this study explores the PHA-induced ER stress response that regulates the antiproliferation and apoptosis of breast cancer cells.

## Data Availability

Data are contained within the article.
